# US MEN’S SHEDS: MAINTAINING PURPOSE AND CONNECTION WITH OTHERS

**DOI:** 10.1093/geroni/igae098.2882

**Published:** 2024-12-31

**Authors:** Melinda Heinz, Kaylin Kent-Thomas, Julie Smith Hinders

**Affiliations:** University of Northern Iowa, Cedar Falls, Iowa, United States; Upper Iowa University, Fayette, Iowa, United States; University of Minnesota Crookston, Crookston, Minnesota, United States

## Abstract

The purpose of this study was to explore U.S. Men’s Sheds, learn how they operate, who attends them, and how participants perceived them. It is the first study exploring Men’s Sheds in the U.S. An explanatory sequential mixed methods study was conducted, and paper-based surveys were sent to sheds registered with the U.S. Men’s Sheds Association. Shed chairpersons encouraged members to complete surveys (N = 76), and results showed that most members were male (91%), older adults (M = 70), in good/excellent health (76%), and joined to make new friends or learn new skills. Four interviews were also conducted and two themes and a subtheme were noted. Theme 1: Purpose of the Men’s Shed represented the varied perspectives on what the Men’s Shed was meant to do for the community. Some members saw it as an extension of the church and felt it was important to share religious perspectives with the community, while others disagreed. A Subtheme on Serving the Community was also noted where members did agree on the importance of serving the community. Theme 2: Building Connections- Possibilities and Challenges illustrated the opportunities Men’s Sheds create for men in/approaching retirement. However, the lack of a formal space for members to meet was a barrier and reduced informal socializing that might otherwise occur. These findings revealed that many of the men joined because they wanted something worthwhile to do with their time. The interviews revealed that there may be opportunities to start Men’s Sheds within existing faith- based organizations.

